# Cultural sensitivity and patient-centered care regarding incense and candle smoke emissions

**DOI:** 10.3389/falgy.2026.1807883

**Published:** 2026-05-01

**Authors:** Gomeo Lam, Cameron Nejat, Mary F. Lee-Wong

**Affiliations:** 1 New York College of Podiatric Medicine of Touro University, Harlem, NY, United States; 2 Rutgers New Jersey Medical School, Newark, NJ, United States; 3 Maimonides Medical Center, Brooklyn, NY, United States; 4 Icahn School of Medicine at Mount Sinai, New York, NY, United States

**Keywords:** air pollutants, asthma, candles, cultural competency, environmental health, incense, religion, respiratory diseases

## Abstract

Incense and candles are commonly utilized for relaxation, deodorization, aesthetics, and religious observance. Both are used ubiquitously and viewed innocuously. However, both modalities release a multitude of compounds, including carbon monoxide, benzene, acrolein, and polycyclic aromatic hydrocarbons, that carry documented risks for cardiovascular disease, pulmonary dysfunction, and cancer. Incense particulate matter generates 45 mg/g vs. 10 mg/g from cigarette smoke. The following two clinical cases illustrate this challenge: an 87-year-old woman with chronic obstructive pulmonary disease and asthma whose acute dyspnea was temporally associated with incense burning during ancestral worship and a 31-year-old atopic asthmatic woman whose symptoms were repeatedly triggered by meditative relaxation through lighting candles. For many patients worldwide, these practices are inseparable from cultural identity and religious devotion, rendering simple cessation counseling insufficient. This article explores the current evidence on health hazards from incense and candle smoke, synthesizes their cultural and religious significance across major traditions, and proposes a structured framework for clinician communication. Practical recommendations, including electronic alternatives, burn duration reduction, improved ventilation, and culturally sensitive phrasing, are presented to help clinicians navigate these conversations effectively. A summary table comparing health risks, cultural roles, and alternative options is provided as a practical clinical reference. Recognizing the cultural and religious significance of incense and candle use across diverse populations is a prerequisite for delivering care that is both clinically effective and respectful of patient values.

## Introduction

Incense and candles are among the most conventional combustion-based products used globally, serving purposes that span lumination, mood enhancement, deodorization, festive celebration, and religious observance. Despite their cultural prevalence, users become inadvertently and unknowingly exposed to the health hazards associated with combustion byproducts, which are frequently underestimated or unknown to users. Simply put, wide-ranging commonplace usage does not forego the inadvertent health risks. Burning these materials generates carbon monoxide, benzene, acrolein, polycyclic aromatic hydrocarbons, and fine particulate matter, which together carry significant risks to respiratory and cardiovascular health (1, 2). Individuals are exposed through direct, firsthand smoke; through secondhand exposure, affecting children and the elderly in shared vicinities; and through thirdhand smoke, i.e., residues that persist on clothing, furniture, and surfaces for months after combustion (3, 4).

Although publications have highlighted numerous health hazards from both incense and candle smoke emissions, these exposures are difficult to eradicate due to the ubiquitous usage ingrained in tradition, lifestyle habits, and religious practices. Recommending cessation without acknowledging this dimension risks undermining the therapeutic alliance and reducing patient adherence to broader health recommendations. Despite the emphasis on patient-centered care in modern clinical discourse, practical guidance for clinicians on how to conduct these sensitive conversations remains sparse in the literature. This perspective addresses that gap by synthesizing the evidence on health hazards, contextualizing the cultural and religious importance of incense and candle use across traditions, and providing actionable clinical recommendations, including example communication strategies and a clinical summary table, for practitioners working in diverse patient populations.

## Methods

A literature search for this perspective article was conducted in PubMed, *JSTOR*, and Google Scholar. Search terms included combinations of the following: “incense smoke,” “candle smoke,” “indoor air quality,” “particulate matter,” “polycyclic aromatic hydrocarbons,” “respiratory disease,” “asthma,” “COPD,” “cardiovascular disease,” “cultural practices,” “religious practices,” and “patient-centered care.” Articles published in English were included, with priority given to studies published from 2010 onward; seminal historical and archaeological references were included regardless of publication date. Studies were included if they addressed chemical composition or health outcomes related to incense or candle smoke, or if they examined cultural, religious, or behavioral dimensions of their use. Review articles, original research, and case reports were all considered. Reference lists of included articles were manually examined for additional relevant sources. The two presented case vignettes were drawn from the authors’ clinical practice and de-identified. These are standard of care examples, where counseling was provided to avoid exposure to noxious triggers that exacerbated respiratory symptoms. This is similar in nature to tobacco cessation counseling and secondhand smoke exposure. All mentions are for illustrative purposes only.

## Case presentations

### Patient 1

An 87-year-old woman with a documented history of chronic obstructive pulmonary disease and asthma who was undergoing supplemental oxygen therapy presented with acute shortness of breath. A disclosed temporal relationship was established between her symptom exacerbations and the burning of incense sticks during traditional ancestral worship rituals at home. When counseled on limiting incense use, she declined, explaining that burning joss sticks was a central expression of veneration and homage for her deceased ancestors: an obligation integral to her cultural and spiritual identity that she was unwilling to forgo.

### Patient 2

A 31-year-old woman with a prior diagnosis of atopic asthma and allergic rhinitis continued burning scented candles as part of her daily relaxation and recreational routine. A direct temporal relationship was inferred between candle smoke exposure and asthma exacerbations, with symptomatology including wheezing, dyspnea, and chest tightness. Although cessation was encouraged, she declined, describing the use of candles as routinely soothing, calming, and meditative.

These cases illustrate a recurring clinical dilemma, i.e., the intersection of documented environmental health risk and deeply personal cultural or lifestyle practice. Both patients demonstrated insight into their diagnoses yet made autonomous decisions to continue the exposures, illustrating the need for a more nuanced clinical approach than straightforward cessation advice.

## Health hazards and environmental impact

### Incense smoke

Incense combustion generates a complex mixture of gaseous and particulate pollutants with systemic health implications. Oxidative stress, uncontrolled cellular proliferation, and cardiovascular disease risk have all been associated with incense exposure (5). Cross-sectional studies have demonstrated an association between chronic incense burning and increased carotid artery intima-media thickness, a validated surrogate marker of atherosclerosis (5). Incense-derived pollutants have also been implicated in insulin resistance and impaired endocrine signaling, with population studies of occupationally exposed incense workers reporting higher rates of prediabetes and type 2 diabetes mellitus (6).

The particulate burden generated by incense is substantially greater than that of cigarette smoke; incense combustion produces approximately 45 mg/g of particulate matter burned, compared with approximately 10 mg/g for cigarette smoke, rendering it among the more potent sources of indoor air pollution in domestic settings (5, 7). In contrast to the dangers of tobacco smoke exposure, incense fume precautions remain understudied and lesser known. Indoor combustion consistently generates higher concentrations of aerosolized particles than outdoor environments; studies of temple settings have measured indoor particulate matter concentrations as high as 626 µg/m^3^ compared with 121 µg/m^3^ outside the same buildings, a four- to six-fold difference (8). Burn duration is also a significant determinant; longer combustion periods produce progressively higher particulate thresholds (9). Beyond particulate matter, conventional incense combustion releases trace metals including lead, chromium, arsenic, and cadmium, which carry independent respiratory and systemic health risks through prolonged inhalation (10).

Studies of adolescent populations have demonstrated associations between household incense exposure and impaired lung function, suggesting that developing respiratory systems are particularly susceptible to indoor incense combustion (11). Younger children, given their proportionally higher respiratory rates and time spent indoors, likely face comparable or greater risk (12). Thirdhand smoke deposited on surfaces further extends exposure beyond the period of active combustion (3, 4). Chronic incense exposure also implicates anatomical brain changes, influencing cognitive resilience and impairing the prevention of cognitive decline, with possible implications for dementia risk (1).

### Candle smoke

Similar to incense, candle burning also has health implications that may outweigh its practical uses. Candle combustion produces fine particulate matter, aromatic amines, aldehydes, and volatile organic compounds; scented candles have been shown to generate higher concentrations of certain gaseous pollutants than unscented varieties, with emission profiles varying substantially depending on fragrance composition and wick type (13). Due to its small size, candle particulate matter is readily inhaled during respiration and accumulates in the alveoli, heightening the risk of cardiopulmonary dysfunction (2). Candle burning has been observed to stimulate indoor microbiome formation, including organisms such as *Pseudomonas* and *Staphylococcus*, which can subsequently disperse through heating, ventilation, and air conditioning systems, furniture, and tactile contact; weather conditions and barometric pressure may further influence the infectious distribution of these organisms (14).

Prolonged candle smoke exposure has also been associated with the risk of urologic malignancy. Carcinogenic aromatic amines in candle smoke can act as ligands for receptors that activate transcriptional cascades, promoting uncontrolled cellular differentiation (15). Despite these hazards, users frequently perceive candles as benign. The association of scented candles with stress reduction and mood enhancement is well-established, and aromatherapeutic compounds have been reported to reduce anxiety and enhance mood, contributing to the psychological benefits users associate with scented candle use (16), which partly explains patients’ resistance to cessation. Patients with chronic respiratory conditions have been documented to use candle burning and aromatherapy as complementary practices, sometimes attributing sacred or natural health significance to these activities in ways that make cessation recommendations particularly challenging to accept (17).

## Cultural and religious context

Despite health considerations, one must also recognize the cultural and religious significance of incense use. The practice is deeper than merely burning a joss stick or curating aromatics. It carries profound cultural and spiritual meaning across many of the world's major traditions, and clinicians who overlook this dimension risk being perceived as dismissive of the patient's deepest-held values. The historical record of incense use spans millennia. In dynastic China, incense symbolized aristocratic status, court etiquette, and benevolence through worship (18). In ancient Egyptian practice, the use of incense was known for its apotropaic representation of malevolent entities and appeasement towards the gods. Paintings would often depict burning sticks as a centered symbolism of holiness and surrender (19). Within Buddhist practice, engaging in these rituals symbolically represented a communicative channel with divinities, with the rising smoke representing prayers ascending to a higher plane (20).

Incense also features extensively in the Abrahamic scriptural tradition (21). Exodus instructs God's mandated priestly obligation of incense usage: “Aaron must burn fragrant incense on the altar every morning when he tends the lamps. He must burn incense again when he lights the lamps at twilight so incense will burn regularly before the Lord for the generations to come” (Exodus 30:7–8, NIV). Similarly, Vayikra (Leviticus) denotes the protective act within liturgical worship, framing incense not as an optional gesture but as a sacred duty:

And he shall place the incense upon the fire, before the Lord, so that the cloud of the incense shall envelope the ark cover that is over the [tablets of] Testimony, so that he shall not die. [Vayikra (Leviticus) 15:13, Torah]

Similarly, many individuals place religious guidance over lifestyle revitalization when managing their overall wellbeing. Christian scripture invokes incense as a symbol of continuous prayer and divine presence. Across these traditions, incense is not merely a cultural artifact but a theologically grounded practice whose discontinuation may, for devout practitioners, carry spiritual consequences.

Candles carry comparably rich symbolic weight. In Christian scripture, candlelight serves as a metaphor for virtue, truth, and divine guidance, imagery that has been internalized across generations of believers for whom lighting a candle is an act of intentional faith: “No man, when he hath lighted a candle, putteth it in a secret place, neither under a bushel, but on a candlestick, that they which come in may see the light” (Luke 11:33, KJV). Beyond formal religion, candles function as instruments of meditation and mindfulness across both secular and contemplative traditions.

Religious and spiritual beliefs have been shown to have a measurable influence on health-related decision-making in culturally diverse populations (22, 23). Clinicians who engage with this reality, rather than treating it as an obstacle to compliance, are better positioned to achieve outcomes that are both medically sound and respectful of patient identity.

With context dependency, rather than solely reproducing extended scriptural quotations, it may be more useful for the practicing clinician to understand the common thematic threads across traditions: incense and candle use across faiths consistently represent a bridge between the human and the sacred, a tangible act of devotion or presence. This shared meaning, regardless of specific tradition, should inform the tone and framing of any clinical recommendation. A summary of health risks, cultural significance, and alternative options for incense and candle use is presented in Table 1.

**Table 1:** Health risks, cultural significance, and alternative options for incense and candle use.

Feature	Incense	Candles
Primary uses	Religious worship, ancestral veneration, meditation, deodorization (3, 18, 20)	Lumination, relaxation, religious ritual, aromatherapy (2, 16, 17)
Key emitted compounds	CO, benzene, acrolein, PAHs, fine particulate matter (4, 5)	CO, aromatic amines, aldehydes, VOCs, fine particulate matter (13, 15)
Particulate burden	∼45 mg/g burned (vs. ∼10 mg/g for cigarette smoke) (5, 7)	Small-diameter particles; tendency toward alveolar deposition (2, 13)
Documented health risks	Asthma exacerbation, CVD, atherosclerosis, endocrine disruption, cognitive decline, cancer (1, 5, 6, 11, 12)	Asthma exacerbation, cardiopulmonary dysfunction, urologic malignancy, indoor microbiome disruption (2, 14, 15)
Cultural/religious significance	Buddhism, Hinduism, Chinese ancestral worship, Judaism, Christianity, Islam (18–21)	Christianity, meditative and secular traditions worldwide (16, 17)
Low-risk alternatives	Electronic aroma devices (spiritually equivalent to conventional burners in Buddhist practice) (20); ceramic flame replicas	Battery-operated LED candles; essential oil diffusers (24)
Culturally sensitive alternative measures	Shorten burn time; improve ventilation; HEPA filtration; spatial distancing (9, 10)	Same; avoid near children, the elderly, and those with respiratory disease (12, 24)

CO, carbon monoxide; CVD, cardiovascular disease; PAH, polycyclic aromatic hydrocarbon; PM, particulate matter; VOC, volatile organic compound; HEPA, high-efficiency particulate air.

## Clinical recommendations

The most effective clinical conversations about incense and candle use begin with curiosity rather than advisement. Before introducing any health information, the clinician should first explore the role these practices play in the patient's life. A non-judgmental, open-ended inquiry establishes rapport and signals respect. Table 2 provides a structured approach.

**Table 2:** Practical communication strategies.

Strategy	Approach
Explore before educating	Invite the patient to describe the meaning and context of their practice before raising health concerns. Patients are measurably more receptive to behavioral health counseling when they feel their values have been acknowledged first.
Apply motivational interviewing principles	Assess the patient's readiness to change using open-ended questions, reflective listening, and affirmations. Avoid prescriptive or directive language implying that total cessation is the only acceptable outcome.
Frame alternatives as additions, not replacements	When introducing electronic or non-combustion alternatives, present them as options that can preserve the ritual's meaning while reducing physical harm, not as substitutes that diminish its spiritual value.
Involve the patient in co-designing a plan	Ask what elements of the practice are most essential (the scent, the flame, the ritual structure, or the communal aspect) to identify where culturally sensitive alternative approaches are most feasible and where non-negotiable elements exist.
Use incremental goals	For patients unwilling to change immediately, small modifications—reduced burn time, improved ventilation, and spatial distancing from sleeping areas—are clinically meaningful and easier to accept as starting points.

The phrases presented in Table 3 may help clinicians open these discussions without sounding judgmental. These templates are designed to demonstrate respect, invite dialogue, and keep the patient as an active partner in decision-making rather than a passive recipient of clinical instruction:

**Table 3:** Recommended phrases for initiating sensitive conversations.

Communication objective	Sample dialogue
Understand the patient's perspective	“I’d like to understand what this practice means to you before we talk about anything else. Can you tell me more about it?”
Inquire about their willingness to learn more about your reasoning	“I can see how central this is to your life. I’d like to share some information about how it may be affecting your breathing. Would that be all right?”
Find a common ground to ensure cultural preservation and health prevention	“I’m not asking you to give up something meaningful. I’d like us to find a way to protect your health while still honoring what you value.”
Understand the patient's overall feeling on moving forward	“Some of my patients have found that a shorter burning time, or using an electronic alternative, allows them to continue the practice while noticing real improvement in their symptoms. Is that something worth exploring together?”
Set structured yet feasible follow-ups	“How would you feel about trying a small modification for a month and seeing how your breathing responds?”

For patients unwilling to fully cease incense or candle use, Table 4 outlines graduated culturally sensitive alternative approaches that are both practical and clinically meaningful.

**Table 4:** Culturally sensitive alternatives.

Alternative	Reasoning
Electronic incense	Aroma-emitting electronic devices that simulate the ritual appearance and scent of burning incense have been evaluated as spiritually valid devotional alternatives among Buddhist practitioners, demonstrating equivalent religiosity and worship intention to conventional combustion-based burners (20).
Reduced burn duration	Since particulate accumulation is proportional to burning time, shortening each session is an accessible modification that reduces exposure without requiring full cessation (9).
Improved ventilation	Open windows, portable air purifiers, or high-efficiency particulate air (HEPA) filtration systems can reduce indoor particulate concentration during and after burning.
Non-combustion candle alternatives	Battery-operated LED candle replicas and ceramic decorative objects simulate the visual and symbolic elements of candlelight without producing combustion byproducts. For patients who agree to cease candle burning, removal of the combustion source has been associated with clinical improvement in candle-related respiratory conditions (24).
Spatial distancing	Burning only in well-ventilated rooms and away from sleeping areas, children, elderly individuals, and those with respiratory conditions reduces secondhand and thirdhand exposure for the most vulnerable household members.

## Limitations

Several limitations of the existing evidence base should be acknowledged. First, most available data derive from cross-sectional and observational study designs, which preclude causal inference regarding the relationship between incense or candle smoke exposure and specific health outcomes; thus, longitudinal and interventional studies are needed. Second, exposure characterization has not been standardized across studies; variability in particle size distribution, chemical composition of combusted materials, and indoor ventilation conditions makes cross-study comparisons difficult and introduces the possibility of exposure misclassification. Third, comparisons between incense, candle, and cigarette emissions, while clinically useful as a reference frame, are frequently drawn from studies employing different measurement protocols, which may introduce systematic bias when quantifying relative risk. Fourth, the case presentations in this article, while clinically instructive, represent individual cases and should not be interpreted as generalizable findings. Individual variation in physiological response and in the cultural or spiritual meaning attached to these practices limits the direct applicability of any single communication framework to all clinical contexts.

## Conclusion

Incense and candle smoke emissions carry documented risks to respiratory, cardiovascular, neurological, and oncological health. For a substantial portion of the global patient population, however, their use is inseparable from cultural identity, ancestral tradition, and religious devotion. The cases presented here demonstrate that patient autonomy and deeply held beliefs frequently supersede clinical advisement—even when patients fully understand the associated risks. Simple cessation counseling, applied without cultural awareness, is both ineffective and potentially harmful to the therapeutic relationship.

As providers, it is imperative to understand the patient's decision-making while prioritizing tailored treatment upon making recommendations. We should engage in a two-way discussion to find balance in honoring religious and cultural practices while preserving one's physiological wellbeing. Clinicians must therefore adopt approaches that are simultaneously evidence-informed and culturally competent. The integration of motivational interviewing, culturally sensitive structured alternative strategies, and electronic or non-combustion alternatives offers a pragmatic and respectful pathway forward. Equipping practitioners with recommended phrases and a structured communication framework—particularly those who may be unfamiliar with the relevant religious or cultural traditions—can meaningfully improve the quality of these clinical encounters. At the policy level, greater public health awareness of indoor air quality risks from domestic combustion sources is warranted, alongside primary care guidelines that specifically address culturally embedded practices.

Future research should prioritize standardized exposure assessment methodologies, longitudinal health outcome studies in populations with habitual incense and candle use, and culturally adapted behavioral intervention trials. Ultimately, honoring the whole patient, i.e., their physiology, their beliefs, and the communities that shape their identity, is not merely a philosophical ideal but the practical foundation of effective, patient-centered care.

## Data Availability

The original contributions presented in the study are included in the article/Supplementary Material, further inquiries can be directed to the corresponding author.
